# Predicting social skills in disadvantaged Chinese high school students through physical education

**DOI:** 10.3389/fpsyg.2023.1149223

**Published:** 2023-04-27

**Authors:** Laura Primo, Juan González-Hernández, Yin Yang, Cristina López de Subijana

**Affiliations:** ^1^Social Sciences Applied to Sport, Physical Activity and Leisure Department, Universidad Politécnica de Madrid, Madrid, Spain; ^2^Department of Personality, Evaluation and Psychological Treatment, Universidad de Granada, Granada, Spain; ^3^School of Psychology, Beijing Sport University, Beijing, China

**Keywords:** rural, China, sport, physical education, young people

## Abstract

**Introduction:**

This study analyzed the relationships between social skills and motivation to Physical Education, students´ perceived support regarding Physical Education lessons (from parents, teachers and peers) and basic needs satisfaction in a sample of disadvantaged high school Chinese students according to Self-Determination Theory-related main principles. Physical Education classes are a great opportunity to develop not only psychomotor and physiologically, but also psychosocially in young people, and that is why this study investigates the relationship between the social skills of the students and these other main variables of the Self-Determination Theory.

**Methods:**

Two hundred and nine disadvantaged students enrolled in a camp organized by a non-governmental organization in Chengdu province (15.9 ± 0.83 years; 73.9% female and 26.1% male) completed the Chinese versions of the following Self-Determination Theory-related questionnaires (independent variables): Learning Climate Questionnaire, Activity- Feeling States Scale, Perceived Locus of Causality scale; and social skills questionnaire (dependent variable): Matson Evaluation of Social Skills with Youngsters Scale.

**Results:**

The standard multiple regression model to predict social skills based on perceived support, basic needs satisfaction and motivation towards Physical Education was significant (*F*(11, 195)= 13.85; *p* < .001; *R^2^*=.44; Cohen’s *f^2^*= 0.78). The social skills of the students were positively related to peer support and relatedness subscales. In contrast, introjected regulation, external regulation, and amotivation were adversely correlated with social skills.

**Discussion:**

We believe that this information would help policymakers and teachers design new policies, actions, and teaching methodologies to implement for the development of Physical Education and sport programs in China, those that will help young people throughout their life span.

## 1. Introduction

### 1.1. Conceptual framework: Self-determination theory (SDT) and social skills in physical education (PE) and sport contexts

Social skills are a person’s capacity to get along with others and to engage in prosocial activity, which impacts their popularity among classmates, teachers, parents, and other relevant adults, which are the skills required for good interpersonal functioning (Matson et al., 2010). Several theoretical perspectives, such as expectancy-value theory (Wigfield and Eccles, 2000), goal theory (Elliot and McGregor, 2001), and SDT, have been used to examine students’ social skills (Ryan and Deci, 2017), but is SDT the one that has been widely used to look at motivation in both educational and PE settings, what is the context of this research, so this is why we chose it as our conceptual framework. Some academics have suggested using PE and sport environments to help young people improve their social skills (e.g., communicational skills, value-based expressions) in terms of developmental objectives (Anderson-Butcher and Bates, 2018; Nascimento Junior et al., 2021) and others also suggest that these abilities will transfer to other life scenarios if they are developed within the environment of PE and sport (e.g., empathy, altruistic behaviors, forgiveness), that fosters social abilities in anticipation (Turnnidge et al., 2014) and protect of antisocial behaviors and social violence (Rodríguez-Franco et al., 2023) when the competitive sport increases. Since among the postulates of the Self-Determination Theory is the promulgation of the relation of motivational constructs and needs to a person’s development and global functioning, including the development of global-life competencies such as social skills (Ryan and Deci, 2017), in this research we wanted to analyzed the relationships between these competencies and Self-Determination Theory main variables in our study context, a sample of disadvantaged Chinese students from rural areas attending PE lessons.

If we delve into the main postulates of SDT, conduct can be explained by self-determination along a continuum of intrinsic motivation, extrinsic motivation, and amotivation. The higher the level of self-determination, the more energy and desire there is to engage in an activity. Increased fulfillment of these needs results in greater self-determination as well (Ryan and Deci, 2017). Deci and Ryan (2013) noted that when the learning environment offers the right kinds of challenges, abundant sources of stimulation, and a context of autonomy, the internal motivational wellspring of learning is likely to blossom, providing students with the chance to become more independent.

On the other hand, three basic psychological needs (autonomy, competence, and relatedness) are essential to energizing human behavior. Due to the necessity of these demands for one’s own development and well-being, people attempt to satisfy them (Ryan and Deci, 2017). A number of positive outcomes arose from providing students with choices. For instance, choosing whom they participated with made students feel more comfortable. Feeling more comfortable and engaging in activities that students perceived to be enjoyable led to increased participation. Providing students with a choice as to what role they undertook (e.g., player, referee, coach) as per the sport education model of teaching, also led to increased perceived autonomy and increased satisfaction of competence as students could select an activity that suited their ability (Knowles et al., 2018). For example, Therefore, having captains who pick teams publicly influenced competence, relatedness, and affective outcomes (White et al., 2021).

To meet fundamental psychological needs and promote self-determination, suitable social conditions for the individual are necessary (Ryan and Deci, 2017), with autonomy support from parents, peers, and teachers defined and assessed as these contexts (Ryan and Deci, 2017). First, according to research, family members—particularly parents—are important influences on children’s participation in physical activity and sports because they provide encouragement and because they share physical activity and sport activities together (Lijuan et al., 2017). According to Lijuan et al. (2017) the family structures, parental roles, and parenting practices in Chinese culture are different from those in western nations (2017). Therefore, it is crucial to look into how parental influences affect children’s PE motivation and participation in physical activity and sport in China. Secondly, peers play a crucial role in determining motivation towards PE since students highly prioritize the importance of having fun with their friends. Friendships not only make PE fun and enjoyable but they also play important roles in buffering against potentially negative aspects of PE (White et al., 2021) been supportive peer relationships linked to relatedness satisfaction, intrinsic motivation, and positive affect. Peers may be the most crucial social element in the PE setting in terms of motivation, need fulfillment, participation, and affective outcomes (McDonough et al., 2013; White et al., 2021). Finally, deepening on the role of teachers, an enthusiastic teacher not only enhances relatedness basic need, but also in-fluences students’ autonomous motivation, and therefore, may in-crease participation in PE (White et al., 2021). School climate can predict kids’ prosocial, antisocial, and violent behavior (McDonough et al., 2013) and PE instructors’ actions will serve as models for and motivators for their students’ actions according to Lund and Kirk (2010).

All in all, as empirically demonstrated in the last paragraphs, greater autonomy support is a key aspect to successfully give students more opportunities for higher self-determined behavior. A substantial body of research has shown that autonomy-supportive circumstances are predictive of greater persistence, deeper internalization of behaviors, values, and attitudes, as well as more adaptable and innovative behavior in PE context (Roth et al., 2011; Manzano-Sánchez et al., 2021).

### 1.2. Deepening social skills in PE and sport contexts

A wide range of disorders across the life span have been proven to be caused by social skills issues; they are often related to social conflicts (e.g., racism, bulling), difficulties with relationships (e.g., love affairs, acceptance of the authority), and even self-esteem and adaptive disorders (e.g., eating disorders, substance abuse) in adolescence (Matson et al., 2010). Therefore, early identification of social skills problems allows for early interventions (Matson et al., 2010). PE gives students the opportunity to engage in moderate-vigorous physical activity, which has commensurate physical and psychological health benefits and is crucial for promoting physical activity in adolescents (Hermens et al., 2017; Rhodes et al., 2017). Furthermore, to encourage children to be physically active not only at school, but also outside of it and throughout their life, PE programs help them preserve habits gained in early ages throughout adulthood, being a crucial factor in the prevention of diseases associated with physical inactivity. PE emphasizes the use of activities to drive communication, cooperation in the group and mutual influence, and the situational characteristics of PE experimental teaching are conducive to the development of individual social skills and prosocial behaviors which are favorable to personality development (Fu and Ding, 2008; Yildiz et al., 2018). It appears that collectively across studies, friendship that you make in PE will make PE class more enjoyable (White et al., 2021), what will allow children to participate more in classes, and thus have more opportunities to further develop their social skills.

Despite the fact that PE and sport can be used to improve social skills, current research indicates that it does not always encourage healthy growth (Gould and Carson, 2008; Durán, 2011): it will depend on how the PE and sport professionals guide the teaching–learning process, being our responsibility to promote opportunities for development of those skills within an arena that occupies so much of our children’s time and attention. PE teachers must place particular emphasis on those behaviors occurring during the game and consequently propose tasks and programs as accurate as possible. The design and implementation of such specific material will undoubtedly lead to interpersonal relationships during the teaching-learning process because it is during this process that interactions mostly concur (Gil-Madrona et al., 2020).

### 1.3. SDT and social skills in PE and sport contexts of China

It is important to mention that, despite the advantages of physical activity mentioned previously, many children in China fall short of the minimum physical activity guidelines (Lu et al., 2017). In secondary schools, all subjects that do not count toward the university entrance exam (*gaokao*) are becoming less important, including PE (Jin, 2016). According to Jin (2016), due to the poor state of the PE curriculum, parents do not care about the growth of students during these PE lessons. Also, the learning interest of the students in the PE class is not very high (Jin, 2016). This downward spiral gradually pushes the present school PE into a marginalized position (Jin, 2016).

Furthermore, China is a large agricultural country and rural children who remain in their village while one or both of their parents with parents working in large cities away from home are referred as “left-behind children.” They have a strong willingness to go to college, but they lack study guidance, professional reporting, and career planning resources to support their future studies. Therefore, they are in a disadvantaged position facing academic and social skills problems (Hu et al., 2020) which affect their developmental trajectories (Taylor and Carlo, 2021). However, new regulations and initiatives are being adopted for the promotion of physical activity and sport in recent years due to the rapid development of China’s economy (Zou, 2022). One of those projects that aims to improve rural areas education is the nongovernmental organization (NGO) with which we are working together in this study.

When we focus on the subject of SDT in sport context in China, we can find some studies focusing on: academic motivation and its relationship with participation in school academic stress enjoyment, Moderate to Vigorous Physical Activity guidelines and intentions to exercise during leisure time (Yang, 2019; Wang and Chen, 2021); impact of coaching methods and sport climate on athletes’ behavior (Fu and Ding, 2008; Chen et al., 2016) and relationship between student’s time practicing physical activity and their behaviors (Wan et al., 2021). Despite the rapid advances in social skills research recently, to date there is not an emphasis of the research on the relation with PE and sport environments, being these studies scarce in Chinese context too.

Consequently, the objective of the current study was to analyze the relationships between social skills and motivation to PE, students’ perceived support regarding PE lesson (from parents, teachers and peers) and basic needs satisfaction in a sample of disadvantaged high school Chinese students according to SDT-related main principles. We hope that the results of this research will be useful to policymakers, educators, and teachers in designing new policies, initiatives, and PE teaching approaches. The hypotheses for our study are featured below:

1. Students’ perceived support would predict their good social skills as the studies made by Cheon et al. (2018), by García-García et al. (2020) and by Trigueros et al. (2020) also supported;
2. Basic needs satisfaction would predict students’ good social skills as the studies made by McDonough et al. (2013), by Roth et al. (2011) and by Trigueros et al. (2020) also supported;
3. Self-determined motivation would predict students´ good social skills and non-self-determined motivation would predict bad social skills of students, as the study of Manzano-Sánchez et al. (2021).

## 2. Materials and methods

### 2.1. Participants

In this cross-sectional study, a total of 209 disadvantaged students from different rural high schools in southwest China in southwest China joined a camp organized by the Chengdu Linyin Public Welfare Service Center (Future China NGO). This NGO organizes summer and winter schools with the aim to help these students to discover their professional interests and expand their knowledge horizons. The camps are usually held in Gulin County (at the junction of southern Sichuan and northern Guizhou provinces), but the last editions were carried out online due to the restrictions caused by the pandemic. We acknowledge the difficulty in accessing this type of participants. Therefore, we applied a purposive sampling to obtain access to the sample. The age range of the sampled students was 15 to 18 (*M* = 15.9, SD = 0.83), being 73.9% female students and 26.1% male students. They had 2.2 PE classes per week (SD = 0.57) of 41.3 min (SD = 5.0).

#### 2.1.1. Measures

##### 2.1.1.1. Perceived autonomy support from parents, teachers, and peers

Student perceptions of social actors’ support for their autonomy (i.e., parents, teachers, and peers) were assessed using the six-item short Chinese version (Zhou et al., 2019) of the Learning Climate Questionnaire (LCQ; Williams and Deci, 1996). The stem of the questionnaire was ‘If I think about PE class’. In total, 18 elements were administered, with 6 elements repeated, each unique to the social agent (e.g., “My teacher gives me the right to make choices”). In total, this questionnaire has 3 factors, each of them being one of our independent variables. LCQ has been used frequently in previous SDT studies and has acceptable validity and reliability (Zhou et al., 2019). The McDonald’s *ω*’s coefficient for the 18 items was 0.94, the *ω*’s for the different factors: 0.91 for teacher autonomy support, 0.92 for parent autonomy support and 0.92 for peer autonomy support.

##### 2.1.1.2. Basic needs satisfaction

The Chinese version was used to gauge how well the students met their three primary psychological demands (Zhou et al., 2017) of the Activity-Feeling States Scale (AFS; Reeve and Sickenius, 1994). The stem of the questionnaire was ‘When I am in PE class’. To evaluate autonomy, competence, and relatedness, nine elements (three for each subscale, each subscale being one of our independent variables) were employed (e.g., “I have good friends close to me”). AFS has been extensively used in previous SDT studies, displaying satisfactory validity and reliability (Zhou et al., 2019). The McDonald’s *ω*’s coefficient for the 9 items was 0.91, which is the *ω*’s for the different factors: 0.81 for autonomy, 0.74 for competence, and 0.79 for relatedness.

##### 2.1.1.3. Student motivation

Using the simplified Chinese version of the Perceived Locus of Causality scale (PLOC; Yang, 2019), the motivation of students for PE was evaluated (Goudas et al., 1994) and five 4-item subscales made up the scale measuring: Intrinsic motivation (e.g., “because PE is fun”), identified regulation (e.g., “because I want to learn sport skills”), introduced regulation (e.g., “because I would feel bad about myself if I did not”), external regulation (e.g., “because that’s the rule”) and amotivation (e.g., “but I really do not know why”), each subscale being one of our independent variables. Students were asked to respond to the stem “I participate in PE lesson…” using a 5-point Linkert scale ranging from 1 to 5 (strongly disagree) to 5 (strongly agree). Prior SDT research has employed the PLOC extensively and found satisfactory validity and reliability (e.g., Sánchez de Miguel et al., 2017). The McDonald’s *ω*’s coefficient for the 20 items was 0.82, being the *ω*’s for the different factors: 0.87 for intrinsic motivation, 0.74 for identified regulation, 0.66 for introjected regulation, 0.66 for external regulation and 0.86 for motivation. Subscales with a reliability below 0.7 were maintained for scale completeness.

##### 2.1.1.4. Students’ social skills

The “Matson Evaluation of Social Skills with Youngsters scale (MESSY),” created by Matson et al. (1983), is one of the most commonly used self-report scales to assess social skills and was the dependent variable of the study. The MESSY scale has been studied with a variety of populations, including those with developmental disabilities, hearing and vision impairment, intellectual disability, mental health disorders, and being appropriate for study in a Chinese society. It was initially created and used to assess the social skills of typically developing children and adolescents (Kee-Lee, 1997). The MESSY scale scores have already been proved to be reliable and valid (Matson et al., 2010). The McDonald’s *ω*’s coefficient for the 62 items was 0.89.

In this study, students´ social skills were assessed using the Chinese version (Kee-Lee, 1997) of the Evaluation of Social Skills with the Youngsters MESSY scale (Matson et al., 1983). The self-report version of the Likert-formatted scale comprises 62 items in its assessment of social behaviors. On the Linkert scale, each response is given a rating between 1 and 5, with 1 denoting “not at all” and 5 denoting “very much.” Four factors emerge from the MESSY report form for students: Factors 1 and 2 are aggressiveness and antisocial behavior, while factors 3 and 4 are pretentiousness and haughtiness and loneliness and social anxiety, respectively.

The MESSY total score is in the direction of negative social skills and is calculated by reversing the ratings for the Appropriate Social Skills subscale and summing the total with the total scale score for the Inappropriate Social Skills subscale. A high MESSY total score indicates poor social skills, whereas a low total score indicates good social skills. *T*-scores can be computed for the subscale scores and the total score (Matson et al., 2010) Later, we recoded the MESSY components to make them easier to grasp., so now a high MESSY total score denotes good social skills.

#### 2.1.2. Procedure

The ethical criteria were met by obtaining a favorable report from the Bioethics Committee of the Universidad Politécnica de Madrid (registration number 2022-012), which allowed this investigation to be carried out. Informed consent was obtained from the NGO camp responsible person and from the parents of the students. Teachers were informed about the project by the main researcher and received instructions on how to distribute the questionnaires. During the camp’s regular class periods, students answered the online questionnaires individually, being the online development of the data collection undertaken synchronous. The average time to answer the questionnaires was 17 min.

#### 2.1.3. Data analysis

With IBM SPSS version 26.0, the validity of each scale, the descriptive and stability statistics of the sample, and Pearson’s correlations between the study variables were determined (IBM Corporation, 2019). To determine the special impact of SDT on students’ social skills, we first looked at the bivariate correlations of the study variables. To determine the unique contribution of SDT to students’ social skills, we performed a standard multiple regression analysis, that is, all the independent variables were entered at the same time (Tabachnick et al., 2013). The criterion variable was a global score of students’ social skills, and the predictor variables were motivation towards PE, perceived support, and basic needs satisfaction. Cohen f indexes the effect size indicators (Cohen, 2013) and the *α*-level was set at.05.

## 3. Results

### 3.1. Preliminary analysis

Table 1 presents the means, standard deviations, correlations, and McDonald’s omegas(‘s) for the study variables. The Mahalanobis distance was used to identify two examples as multivariate outliers with *p* < 0.001 and they were removed, leaving 207 cases. With this sample, the *post hoc* power was.97 for large effect sizes at *α* = 0.05. The correlations of all variables revealed a significant connection (*p* < 0.01).

**Table 1:** Descriptive statistics and correlations of study variables.

	*M ± SD*	Scale	1	2	3	4	5	6	7	8	9	10	11	12
1. Social skills	1.96 ± 0.21	1–5	*0.89*	0.302^**^	0.477^**^	0.330^**^	0.333^**^	0.288^**^	0.459^**^	0.225^*^	0.199^*^	−0.327^**^	−0.445^**^	−0.488^**^
2. Parents perceived support	3.75 ± 0.86	1–5		*0.92*	0.462^**^	0.571^**^	0.463^**^	0.445^**^	0.489^**^	0.283^**^	0.335^**^	0.023	−0.139^*^	−0.238^**^
3. Peers perceived support	4.04 ± 0.68	1–5			*0.92*	0.577^**^	0.472^**^	0.484^**^	0.615^*^	0.371^**^	0.392^**^	−0.040	−0.224^*^	−0.296^**^
4. Teachers perceived support	3.88 ± 0.75	1–5				*0.91*	0.551^**^	0.413^**^	0.579^**^	0.500^**^	0.493^**^	0.067	−0.203^*^	−0.330^**^
5. Autonomy	3.62 ± 0.80	1–5					*0.81*	0.720^**^	0.679^**^	0.374^**^	0.360^**^	−0.011	−0.250^**^	−0.312^**^
6. Competence	3.71 ± 0.74	1–5						*0.74*	0.663^**^	0.403^**^	0.375^**^	−0.074	−0.224^*^	−0.333^**^
7. Relatedness	3.69 ± 0.77	1–5							*0.79*	0.380^**^	0.356^**^	−0.043	−0.201^*^	−0.347^**^
8. Intrinsic motivation	4.02 ± 0.80	1–5								*0.87*	0.790^**^	0.104	−0.248^**^	−0.444^**^
9. Identified regulation	3.80 ± 0.73	1–5									*0.74*	0.334^**^	−0.028	−0.301^**^
10. Introjected regulation	2.88 ± 0.89	1–5										*0.66*	0.592^**^	0.465^**^
11. External regulation	2.63 ± 0.79	1–5											*0.66*	0.663^**^
12. Amotivation	2.08 ± 0.93	1–5												*0.86*

*M* = Mean; SD = Standard deviation; in italics the Mc Donald’s Omega. ^*^p < 0.05; ^**^p < 0.01.

On average, (1) students had high levels of intrinsic motivation, moderate-high levels of identified regulation, moderate levels of introjected regulation, moderate levels of external regulation, and moderate-low levels of amotivation, mainly self-determined; (2) moderate-high levels of perceived autonomy, competence, and relatedness competence; (3) moderate-high levels of perceived autonomy support from teachers and parents, and high level from friends; and (4) moderate level of social skills.

When examining the relationships between the five motivational regulations and social skills, intrinsic motivation and identified regulations were positively and poorly correlated with good social skills (*r* = 0.20–0.23, *p* < 0.05). In contrast, introjected regulation, external regulation, and amotivation were negatively and low-moderate correlated to good social skills (*r* = 0.33–0.49, *p* < 0.01). Also, the three basic psychological needs were positively and low-moderate correlated with good social skills (*r* = 0.33–0.46, *p* < 0.01) and the students’ perceived support from the three agents were positively and low-moderate correlated with good social skills (*r* = 0.33–0.48, *p* < 0.01).

### 3.2. Main analysis

The findings of the standard multiple regression analysis to predict social skills based on students´ perceived support, basic needs satisfaction, and motivation to exercise are shown in Table 2, and were significant (*F*(11, 195) = 13.85; *p* < 0.001; *R*^2^ = 0.44; Cohen’s *f*^2^ = 0.78). The adjusted *R*^2^ value of 0.41 showed that nearly half of the SDT subscales were able to forecast some of the variability in student social skills. 95% confidence intervals were constructed for the regression coefficients that were significant. From the perceived support of the social agents’ subscales in relation to the PE lesson, the peers’ support (0.037; 0.126) was significant. From the basic needs satisfaction subscales, relatedness (0.028; 0.121) was significant while competence was close to reaching significance (*p* = 0.053). From the motivation towards PE subscales, introjected regulation (−0.750; −0.001), external regulation (−0.088; −0.003), and amotivation (−0.080; 0.006).

**Table 2:** Standard multiple regression results.

DV: Social skills	Unstandardized coefficients		Standardized coefficients		
	*B*	*SE*	*β*	*p*	*sr^2^* (unique)
Parents perceived support	0.013	0.017	0.055	0.43	
Peers perceived support	0.082	0.023	0.269	0	0.04
Teachers perceived support	−0.017	0.023	−0.061	0.46	
Autonomy	0.01	0.023	0.04	0.65	
Competence	−0.047	0.024	−0.169	0.05	0.01
Relatedness	0.074	0.024	0.277	0	0.03
Intrinsic motivation	−0.042	0.025	−0.16	0.1	
Identified regulation	0.049	0.029	0.17	0.09	
Introjected regulation	−0.038	0.019	−0.163	0.04	0.01
External regulation	−0.045	0.022	−0.172	0.04	0.01
Amotivation	−0.043	0.019	−0.193	0.02	0.01
					*R^2^* = 0.44
					Adjusted *R*^2^ = 0.41
					*R* = 0.66^**^

^**^p < 0.01. Unique variability = 0.11; shared variability = 0.33%. DV = dependent variable; *SE* = standard error; *sr*^2^ (unique) = semipartial correlations.

Together, these five predictor factors made a contribution of 0.11 in distinctive variation (semipartial correlations), however peer support (*sr^2^* = 0.04) and relatedness (*sr*^2^ = 0.03) contributed more notably. The 11 predictor factors collectively predicted 33% (30% adjusted) of the (shared) variability in students’ social abilities.

The size and direction of the coefficients indicated a favorable relationship between the relatedness and peer support subscales and the total student social skills. Social skill was negatively related with introjected regulation, external regulation, and amotivation subscales. Because all tolerance values were over 0.10 and the variance inflation factor (VIF) values for each predictor variable were below 4, multicollinearity was ideal for the standard multiple regression analysis (Hair et al., 2014).

## 4. Discussion

This study examined how social skills of students are predicted by their motivation towards PE, basic needs satisfaction, and perceived support. The overall model for predicting students’ social skills based on their desire for PE, basic needs satisfaction, and perceived support is relevant to the study’s goal. On the one hand, the support from their peers and the satisfaction of the relatedness need help developing social skills. On the other hand, controlled types of motivation and amotivation buffer building good social skills. This study has strengths worthy of consideration because there are few studies that study the relationship between SDT and social skills in a sport context in China. They will describe the relevance of our findings that replicate and expand on previous research outcomes. Then, we will propose practical implications and highlight the limitations of this research in the following sections.

Regarding the general correlations, the significant relationship between SDT variables confirm previous studies within this framework where perceived support from teacher, peers, and parents, the three basic needs satisfaction and self-determined motivation were positively related with good social skills (Fu and Ding, 2008; Hodge and Lonsdale, 2011; Chen et al., 2016; Nascimento Junior et al., 2021).

The results of the standard multiple regression partially supported the first hypothesis in which students’ perceived support would predict their good social skills. The present study suggests greater evidence that favors the role of perceived support from peers in relation to the PE lesson, not being significant for regression neither perceived support from teachers nor from parents. According to McDonough et al. (2013) and White et al. (2021), was the perceived support from peers dimension also the one that contributed more to good social skills, suggesting that peers relationships have been linked to relatedness satisfaction, intrinsic motivation, and positive affect, and indicating that peers may be the most crucial social component of the PE environment for motivation, need fulfillment, participation, and affective outcomes. However, other studies suggest also that school climate also could predict and discriminate prosocial and antisocial behaviors and violence (Gano-Overway, 2013; Mayfield et al., 2017; Jang et al., 2020). According to Lund and Kirk (2010) instructors (e.g., PE teachers and monitors) may improve students’ emotional and cognitive skills through PE, being those behaviors they model and encourage the ones on which students will base themselves to carry out their actions, shaping their behavior. Chen et al. (2016), also more focus in a sports competitive context rather than in an educational one, also supported that coaching methods will predict Chinese athletes’ prosocial and antisocial behavior. In discussing the findings of the dimension of parents´ support, it should be noted from the outset that there was a high number of students who were left behind children, so they did not spend a lot of time with their parents at home, which largely nullifies the face-to-face relationships between the two, and perhaps it is one of the reasons why this factor is not contributing to good social skills.

Another interesting finding of this study are the results of the second hypothesis, in which basic needs satisfaction would predict the good social skills of students. We could partially confirm this hypothesis since results suggest that relatedness satisfaction was the most important basic needs satisfaction for predicting good social skills. This result supports the theoretical hypothesis made by Hodge et al. (2016) that satisfying fundamental needs is related to the acquisition of life skills through sport. A sense of connection and belonging in a social setting is referred to as relatedness need (Ryan and Deci, 2017), and it seems that peer relatedness may be a superior basic need during adolescence, as during those ages students will choose to build strong relationships, in contrast to relationships with adults (parents or teacher). In a more competitive context more than educational one, Fu and Ding (2008) also supported the idea that sport climate influences Chinese players’ development of social skills. Neither autonomy nor competence were significant predictors of good social skills. The desire for autonomy is defined as having the power to make decisions and take responsibility for one’s own actions (Ryan and Deci, 2017), but far too frequently, parents, educators, and politicians have ignored this issue and seen education as an external process that must be pushed and prodded from outside (Deci and Ryan, 2013), not letting students develop autonomy competence. Competence need is the ability to feel in control and provide predictable results so that you can work effectively with your environment (Ryan and Deci, 2017), also not significant predictor of good social skills in this study. However, there are still more studies to be conducted to confirm the relationship of the three basic needs satisfaction with the development of social skills in PE and sport context.

And finally, in relation to our third hypothesis, in which the self-determined motivation would predict students’ good social skills and non-self-determined motivation in PE would predict bad social skills, the results partially supported the hypothesis too. Non-self-determined motivation dimensions (introjected, external regulation and amotivation) predicted bad social skills. Performing PE as an obligation or as a reward buffers the development of good social skills. Therefore, PE lessons should avoid promoting these types of motivation. The freedom of choice could be given to some content or exercises without losing the main goal of the lesson. A fundamental characteristic of human functioning is curiosity; people are naturally curious and want to learn new things, and this curiosity could be a major driving force behind educational progress. But far too often, educators, parents, and legislators have disregarded inner drive and seen learning as an extrinsic process that needs to be pushed and prodded from outside (Deci and Ryan, 2013). Although we did not find any self-determined dimension significant in the model, previous studies, like Hodge and Lonsdale (2011) study, found that self-determined motivation was a reliable indicator of teammates’ prosocial behavior. As Deci and Ryan (2013) highlighted, this kind of motivation is a form of drive that originates from an individual’s interest or enjoyment rather than from external demands or rewards, the higher the level of intrinsic motivation, the more energy and desire there is to engage in an activity.

## 5. Practical implications and future research

The results of the study have significant implications. First and foremost, in the late adolescence of disadvantaged students from southwestern rural China, the perception they have of the support of their peers is very important on developing good social skills. In this sample, since many of the students are left-behind children and they do not live with their parents, the perceived support of their parents to PE lesson is not predicting students’ social skills. The perceived support from PE teachers is not a predictor of good social skills either in this context, so maybe new teaching methods for school PE are needed to further develop social skills in students. In this sense, Jin (2016) also noted this is a need within the current school PE system in China. All in all, the peers support to PE is a key feature to develop good social skills on these students, helping them to build strong relationships on this period of life. The relatedness satisfaction will be also very important in this stage of life for these kinds of students, being peer relatedness a superior basic need during adolescence since during those years, kids will choose to form lasting relationships with their peers rather than with adults.

Furthermore, it is important to highlight that non-self-determined PE motivation was a significant predictor of bad social skills. Too frequently, educators, parents, and politicians have ignored intrinsic motivation and seen learning as an extrinsic process that requires external pressure (Deci and Ryan, 2013), but according to this study external rewards should be controlled as they are not only contributing to the development of good social skills, but contributing to the development of bad social skills.

The results of the study can teach us what is the current situation of the disadvantaged students of this area of rural China in relation to PE, their motivations and their needs, and how these aspects can contribute to the development of their social skills (Cheon et al., 2019). It is crucial to remember that social skills serve as the cornerstone of harmony, peace and collaboration (Taylor and Carlo, 2021) what becomes even more important for the millions of children who are born into and nurtured in risky and adverse situations like the sample of our study. As we have seen during this manuscript, PE is a fundamental factor that can affect the development of these social skills, sometimes improving them or even worsening them in other situations, depending on the situation of each student (Cheon et al., 2018). PE places a strong emphasis on the use of activities to promote communication, group cooperation, and mutual influence, and the situational elements of PE experimental teaching are beneficial for the development of individual social skills and prosocial behaviors that are beneficial for personality development and for daily life activities (Bruner et al., 2014; Yildiz et al., 2018; Balçikanli et al., 2019), as well as for their development as individuals in an integral way.

It is important to notice that many young people in China do not even reach the Moderate to Vigorous Physical Activity minimum guidelines, which is even more difficult in rural areas due to the lack of resources they have (Liu and Li, 2017), what we consider a serious problem due to all the aforementioned implications that this can cause. We hope that the results of this study will be useful in helping politicians create new initiatives to put into place for the development of PE and sport programs in China.

With our study, we are contributing to the development of this area of research, opening the way for future researchers to continue on this line based on more scientific studies. For future research, it is important to emphasize the role of PA in the development of social skills and long-term health of young people (Turnnidge et al., 2014; Wan et al., 2021); this will undoubtedly be a topic worth studying in depth in the future.

## 6. Limitations

Despite being a considerable sample, it is important to point out that it is collected from only one NGO, understanding this situation as one of the major weaknesses of the study. In addition, the use of purposive (non-probability) sampling to gain access to the sample and some structural issues for access to the sample (e.g., territorial dispersion, commitment of families to school participation). Not being a representative sample number for rural PE across the country, it represents an example of what the situation is in this area of China, at the confluence of southern Sichuan and northern Guizhou provinces, and probably the situation is similar in other areas of rural China with students of these characteristics (being something to be explored in future research efforts).

In addition, the camps of the NGO China Future (where face-to-face different dynamics of play and psychoeducation with children are established), some complementary actions have been carried out in the last editions due to the restrictions caused to the COVID-19 pandemic, so some data have been collected through online questionnaires. Furthermore, and although the design of the present study aims to provide preliminary information on those links that can be established between PE and the development of prosocial resources, its cross-sectional nature should not establish a causal relationship, but rather some intuitive trends to continue efforts to improve the educational, social and individual quality of children in rural areas of China.

## 7. Conclusion

In summary, we conducted a SDT-based motivational model in which we hypothesized which SDT variables in PE context better predict social skills of students, in this case, disadvantaged students of rural China. We found that the ability to establish relationships with their peers is the most important contribution to promote good social skills. PE teachers should avoid external rewards during PE lessons that seem to stimulate bad social skills.

In general, the research revealed a wide range of themes that broaden our understanding of the effects of sport-based youth development programs, particularly those that target youth of disadvantaged backgrounds in rural China. The results might also be applicable to more extensive youth development programming. For further research we will make comparisons between different populations in China or between China and other countries students in order to analyze how the social skills are developed by students. We believe this information would help policymakers to design new policies and actions to implement for the development of PE and sport programs in China, those that will help students throughout their life span.

## Data availability statement

The raw data supporting the conclusions of this article will be made available by the authors, without undue reservation.

## Ethics statement

The studies involving human participants were reviewed and approved by The Bioethics Committee of the Universidad Politécnica de Madrid (registration number 2022-012). Written informed consent to participate in this study was provided by the participants' legal guardian/next of kin.

## Author contributions

LP: conceptualization, development of research tools, data collection, methodology, original draft writing, and writing—review and editing. CL: data analysis, methodology, original draft writing, and writing—review and editing. JG and YY: conceptualization, formal analysis, visualization, and writing—review and editing. All authors contributed to the article and approved the submitted version.

## Funding

This work was supported by the “SEED fund” of the Universidad Politécnica de Madrid (VSEMILLA22GLS).

## Conflict of interest

The authors declare that the research was conducted in the absence of any commercial or financial relationship that could be construed as a potential conflict of interest.

## Publisher’s note

All claims expressed in this article are solely those of the authors and do not necessarily represent those of their affiliated organizations, or those of the publisher, the editors and the reviewers. Any product that may be evaluated in this article, or claim that may be made by its manufacturer, is not guaranteed or endorsed by the publisher.
